# Supplementary material for: Contrastive learning of heart and lung sounds for label-efficient diagnosis
Source: Patterns (N Y). 2021 Dec 7;3(1):100400. doi: 10.1016/j.patter.2021.100400 (PMC8767307; doi:10.1016/j.patter.2021.100400)
Supplement: Supplementary file 1 — Document S1. Tables S1–S3 [file mmc1.pdf]

Patterns, Volume *3*

Supplemental information

Contrastive learning of heart and lung sounds for label-efficient diagnosis

Pratham N. Soni, Siyu Shi, Pranav R. Sriram, Andrew Y. Ng, and Pranav Rajpurkar

Supplementary Tables

Table S1: Supplementary data for Figure 2.

		AUC (95% CI)	
Type	Augmentation	10% fraction	100% fraction
No Encoder Baseline	No Augmentation	0.664 (0.630, 0.694)	0.803 (0.755, 0.841)
Supervised Learning	No Augmentation	0.773 (0.737, 0.806)	**0.930 (0.904, 0.954)**
Supervised Learning (full)	No Augmentation	**0.889 (0.865, 0.913)**	0.929 (0.907, 0.949)
	Spectrogram	0.756 (0.728, 0.783)	0.920 (0.894, 0.948)
	Split	0.797 (0.768, 0.832)	**0.927 (0.901, 0.952)**
	Spectrogram + Split	0.787 (0.753, 0.827)	0.924 (0.898, 0.952)
	Frequency Only	0.792 (0.764, 0.817)	0.925 (0.900, 0.950)
Self-supervised Learning (finetune)	Time Only	**0.857 (0.828, 0.885)**	**0.927 (0.901, 0.951)**
	Spectrogram	0.660 (0.619, 0.694)	0.766 (0.719, 0.805)
	Split	0.744 (0.711, 0.778)	0.807 (0.763, 0.850)
	Spectrogram + Split	0.752 (0.715, 0.791)	0.795 (0.759, 0.836)
	Frequency Only	0.666 (0.663, 0.696)	0.782 (0.736, 0.818)
Self-supervised Learning (linear)	Time Only	**0.808 (0.772, 0.838)**	**0.874 (0.841, 0.905)**

Table S2: Supplementary data for Figure 3.

		AUC (95% CI)	
Type	Augmentation	10% fraction	100% fraction
No Encoder Baseline	No Augmentation	0.512 (0.484, 0.536)	0.516 (0.463, 0.559)

Supervised Learning	No Augmentation	0.628 (0.585, 0.673)	0.690 (0.636, 0.754)
Supervised Learning (full)	No Augmentation	**0.687 (0.636, 0.730)**	**0.710 (0.655, 0.774)**
	Spectrogram	**0.633 (0.582, 0.697)**	**0.691 (0.628, 0.758)**
	Split	0.562 (0.510, 0.618)	0.584 (0.519, 0.645)
	Spectrogram + Split	0.562 (0.514, 0.615)	0.650 (0.586, 0.718)
	Frequency Only	0.618 (0.568, 0.671)	0.671 (0.615, 0.734)
Self-supervised Learning (finetune)	Time Only	0.627 (0.572, 0.685)	0.627 (0.571, 0.690)
	Spectrogram	**0.652 (0.597, 0.704)**	**0.659 (0.600, 0.716)**
	Split	0.558 (0.528, 0.584)	0.552 (0.499, 0.603)
	Spectrogram + Split	0.533 (0.498, 0.568)	0.609 (0.549, 0.668)
	Frequency Only	0.649 (0.601, 0.684)	0.656 (0.597, 0.713)
Self-supervised Learning (linear)	Time Only	0.643 (0.598, 0.695)	0.654 (0.595, 0.715)

Table S3: Supplementary data for Figure 4.

		AUC (95% CI)	
Type	Pair Selection Method	10% fraction	100% fraction
	Pos. Same Loc.	0.678 (0.621, 0.735)	0.692 (0.635, 0.758)
	Pos. Dif. Loc.	0.681 (0.626, 0.745)	0.702 (0.643, 0.764)
	Pos. Same Loc./Neg. Same Loc.	0.732 (0.679, 0.783)	0.768 (0.717, 0.818)
Self-supervised Learning (finetune)	Pos. Sim. Age	0.665 (0.610, 0.720)	0.695 (0.638, 0.752)

	Neg. Sim. Age	0.782 (0.737, 0.830)	0.785 (0.734, 0.838)
	Neg. Sim. Sex	0.754 (0.703, 0.806)	0.765 (0.711, 0.824)
	Neg. Sim. Age + Sex	**0.822 (0.782, 0.854)**	**0.842 (0.803, 0.876)**
	Pos. Same Loc.	0.690 (0.638, 0.736)	0.700 (0.641, 0.757)
	Pos. Dif. Loc.	0.681 (0.635, 0.733)	0.689 (0.636, 0.747)
	Pos. Same Loc./Neg. Same Loc.	0.695 (0.646, 0.742)	0.745 (0.691, 0.796)
	Pos. Sim. Age	0.663 (0.610, 0.713)	0.674 (0.618, 0.726)
	Neg. Sim. Age	0.788 (0.739, 0.834)	0.773 (0.718, 0.820)
	Neg. Sim. Sex	0.723 (0.675, 0.770)	0.748 (0.692, 0.804)
Self-supervised Learning (linear)	Neg. Sim. Age + Sex	**0.854 (0.823, 0.882)**	**0.863 (0.834, 0.890)**
