# Supplementary material for: Contrastive learning of heart and lung sounds for label-efficient diagnosis
Source: Patterns (N Y). 2021 Dec 7;3(1):100400. doi: 10.1016/j.patter.2021.100400 (PMC8767307; doi:10.1016/j.patter.2021.100400)
Supplement: Document S2. Article plus supplemental information [file mmc2.pdf]

Article

Patterns

Contrastive learning of heart and lung sounds for label-efficient diagnosis

Graphical abstract

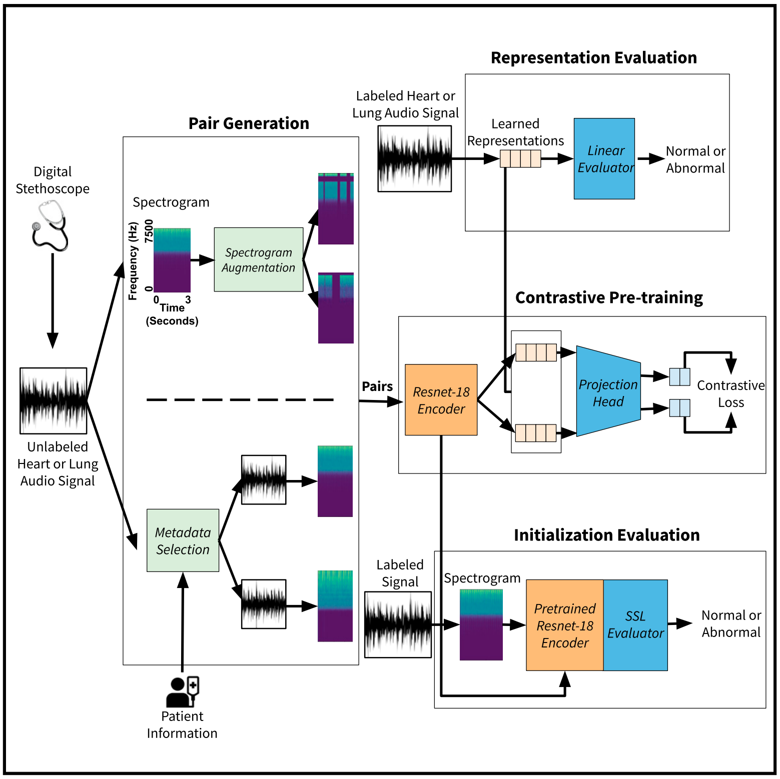

Highlights

- Contrastive learning uses unlabeled data to learn representations
- A new contrastive learning framework is proposed for metadata pair selection
- We show its application in medical heart and lung sound data and metadata
- The contrastive learning strategy only needs 10% of labeled training data

Authors

Pratham N. Soni, Siyu Shi, Pranav R. Sriram, Andrew Y. Ng, Pranav Rajpurkar

Correspondence

siyushi@stanford.edu (S.S.), pranav_rajpurkar@hms.harvard.edu (P.R.)

In brief

The project focuses on a common problem in machine learning in specialized fields: a lack of labeled data. We propose a solution with a contrastive learning framework to leverage unlabeled data and use associated metadata for pair selection to learn data representations. With this framework, we are able to improve downstream tasks performances with limited labeled data. We choose to apply this framework in healthcare, where unlabeled data are abundant and data labeling is expensive.

Soni et al., 2022, Patterns *3*, 100400
January 14, 2022 © 2021 The Author(s).
https://doi.org/10.1016/j.patter.2021.100400

CellPress

Patterns

CellPress
OPEN ACCESS

Article

Contrastive learning of heart and lung sounds for label-efficient diagnosis

Pratham N. Soni,[1] Siyu Shi,[2,4,*] Pranav R. Sriram,[1] Andrew Y. Ng,[1] and Pranav Rajpurkar[3,5,6,*]
[1]Department of Computer Science, Stanford University, Stanford, CA, USA
[2]School of Medicine, Stanford University, Stanford, CA, USA
[3]Department of Biomedical Informatics, Harvard Medical School, Boston, MA, USA
[4]Twitter: @siyushimd
[5]Twitter: @pranavrajpurkar
[6]Lead contact
*Correspondence: siyushi@stanford.edu (S.S.), pranav_rajpurkar@hms.harvard.edu (P.R.)
https://doi.org/10.1016/j.patter.2021.100400

THE BIGGER PICTURE Annotating data at scale is time consuming, especially in specialized domains, such as healthcare, agriculture, and autonomous driving. The scarcity of labeled data can limit the effectiveness of supervised learning. In contrast, there is usually access to more unlabeled data. Unlabeled data can be used through unsupervised learning. One type of unsupervised learning is self-supervised learning, where representations of data are learned from unlabeled data through pretext tasks and are later used for supervised learning tasks. We propose a new contrastive learning framework that leverages metadata in selecting pairs during contrastive learning. We demonstrate the application of the framework in diagnosing heart and lung diseases through heart and lung sound recordings and associated clinical metadata. Our strategy could also be applied in other medical settings such as electronic health records and medical imaging, as well as beyond medicine.

1 2 3 4 5 **Proof-of-Concept:** Data science output has been formulated, implemented, and tested for one domain/problem

SUMMARY

Data labeling is often the limiting step in machine learning because it requires time from trained experts. To address the limitation on labeled data, contrastive learning, among other unsupervised learning methods, leverages unlabeled data to learn representations of data. Here, we propose a contrastive learning framework that utilizes metadata for selecting positive and negative pairs when training on unlabeled data. We demonstrate its application in the healthcare domain on heart and lung sound recordings. The increasing availability of heart and lung sound recordings due to adoption of digital stethoscopes lends itself as an opportunity to demonstrate the application of our contrastive learning method. Compared to contrastive learning with augmentations, the contrastive learning model leveraging metadata for pair selection utilizes clinical information associated with lung and heart sound recordings. This approach uses shared context of the recordings on the patient level using clinical information including age, sex, weight, location of sounds, etc. We show improvement in downstream tasks for diagnosing heart and lung sounds when leveraging patient-specific representations in selecting positive and negative pairs. This study paves the path for medical applications of contrastive learning that leverage clinical information. We have made our code available here: https://github.com/stanfordmlgroup/selfsupervised-lungandheartsounds.

INTRODUCTION

Data labeling is an expensive and time consuming process in machine learning. This problem is exacerbated in domains where trained experts are required to label data, such as agriculture, healthcare, and language translation. Supervised learning models, which rely on labeled data for generalization, thus encounter a bottleneck when obtaining large amounts of labeled data is prohibitive.

Contrastive learning, a type of SSL, is a potential solution to the problem of limited labeled data by using unlabeled data to learn general representations of data, contrasting similar

This is an open access article under the CC BY license (http://creativecommons.org/licenses/by/4.0/).

(positive) and dissimilar (negative) pairs of examples.[1] Employment of contrastive learning has shown powerful results through applications in imaging,[2] video,[3] audio,[4,5] etc. One way to generate a positive pair of examples is through augmentation. The quality and choice of augmentation influences whether models can learn good representations.[6] It has been observed that augmentation methods used for computer vision may not perform well for signal data such as electrocardiograms.[2] These results prompt exploration of optimal augmentation methods specific for each type of data, as well as experimentation with frameworks that leverage metadata to improve learned representations. Previous work has explored methods to leverage metadata associated with unlabeled data in SSL, including encoding genre and playlist associated with song audio for song representation,[5] using patient metadata associated with ultrasound as weak labels,[7] and selecting contrastive pairs based on patient and study information.[8]

Here, we propose a contrastive learning framework that utilizes unlabeled data with additional associated information for selecting contrastive pairs. We apply the framework on heart and lung sounds. Specifically, our method uses audio and spectrogram augmentation on unlabeled heart and lung sounds, with the downstream task of classifying diseases using heart and lung sounds. We further explore the use of clinical information including age group, sex, and recording location to create positive and negative pairs of examples and to leverage insights from clinical information associated with the recordings. We show that using age group (adult versus children), sex, and performance, measured with area under the receiver operating characteristic (AUROC) (area under the ROC curve [AUC]), increases to 0.854 (95% confidence interval [CI]: 0.823, 0.882) and 0.863 (95% CI: 0.834, 0.890), compared with baseline AUCs of 0.512 (95% CI: 0.484, 0.536) and 0.516 (95% CI: 0.463, 0.559), when using 10% and 100% of labeled training data, respectively. These results demonstrate the potential of contrastive learning, especially when leveraging associated clinical information in medicine, including future applications in video monitoring, home monitoring,[9] health records,[10] medical imaging, and even beyond medicine, including vehicle identification and autonomous driving,[11] photometric plant phenotype estimation in agriculture,[12] speech recognition,[13] and text characterization.[14]

RESULTS

Framework for evaluating contrastive schemes

Through contrastive learning, we are able to pre-train an encoder backbone (pre-trained model) to provide feature representations of the initial input data. We can either use the resultant feature representations for inference or further fine-tune the pre-trained model with the addition of a two-layer SSL evaluator using labeled data. Details of the SSL evaluator are described in the experimental procedures. To measure the performance of contrastive schemes, we consider two tasks: How well does the pre-trained model generate latent representations of the data and how good are the pre-trained weights for initialization before fine-tuning? We compare the results of SSL to linear baselines and supervised learning to establish performance comparisons for representations and initializations, respectively. We train and evaluate these methods on two datasets: the respiratory sound database for lung sounds[15] and the PhysioNet Heart Challenge for heart sounds.[16] The databases are labeled with diseases and demographic information. For each dataset, we evaluate models with 10% and 100% of the labeled training data.

Augmentation-based contrastive learning

As a baseline, we examine whether our contrastive learning methodology can better leverage limited labeled data needed compared to supervised learning through three experimental setups using two different fractions of labeled data (10% or 100%) in each. For generating contrastive views, we use augmentations to hide spectrogram information (time and frequency dimensions) from the model. We consider the following augmentation schemes: splitting, time masking, frequency masking, spectrogram masking, and spectrogram masking and splitting. With splitting, we slice a contiguous section from the sample with a time duration of half of the original. Time and frequency masking zero out bands of values along the respective dimensions in the non-zero portion of the sample. Spectrogram masking consists of applying both frequency and time masking. We also consider spectrogram masking and then splitting. Examples of augmentations are shown in Figure 1.

Evaluating representations via linear evaluation

When examining representations with linear evaluations, we observe baseline AUCs of 0.664 (95% CI: 0.630, 0.694) and 0.803 (95% CI: 0.755, 0.841) when using 10% and 100% of labeled training data, respectively. We note that learned representations from unlabeled data are dependent on the augmentation scheme utilized. At both 10% and 100% data levels, masking along the time dimension alone provides the strongest boost ($p < 0.001$ and $p < 0.001$, respectively) in performance, with AUC levels of 0.808 (95% CI: 0.772, 0.838) and 0.874 (95% CI: 0.841, 0.905). Statistical significance is measured against the second-best augmentation (splitting), using a two-tailed two-sample paired t test. Splitting and spectrogram masking and splitting are also effective at improving performance in the limited data regime (AUCs of 0.744 [95% CI: 0.711, 0.778] and 0.752 [95% CI: 0.715, 0.791] at 10%, respectively). On the other hand, spectrogram masking and frequency masking alone do not show significant improvement.

Evaluating initializations via end-to-end fine-tuning

For initializations, we record baseline performance of 0.773 (95% CI: 0.737, 806) and 0.930 (95% CI: 0.904, 0.954) at 10% and 100%. We find that, again, masking along the time axis provides significant improvement ($p < 0.001$) in performance at the 10% level, approaching supervised performance on the full pre-train split (0.857 [95% CI: 0.828, 0.885] versus 0.889 [95% CI: 0.865, 0.913]). None of the remaining augmentations match supervised performance. We further observe that at 100% training data, all schemes provide comparable performance with no significant difference in performance (0.925 [95% CI: 0.900, 0.950] for frequency masking, for example).

We present experimental testing results for heart sound classification in Figure 2.

Contrastive learning for lung sounds

Evaluating representations via linear evaluation

In order to evaluate representations learned from the contrastive learning framework, we find that all contrastive schemes provide

CellPress
OPEN ACCESS

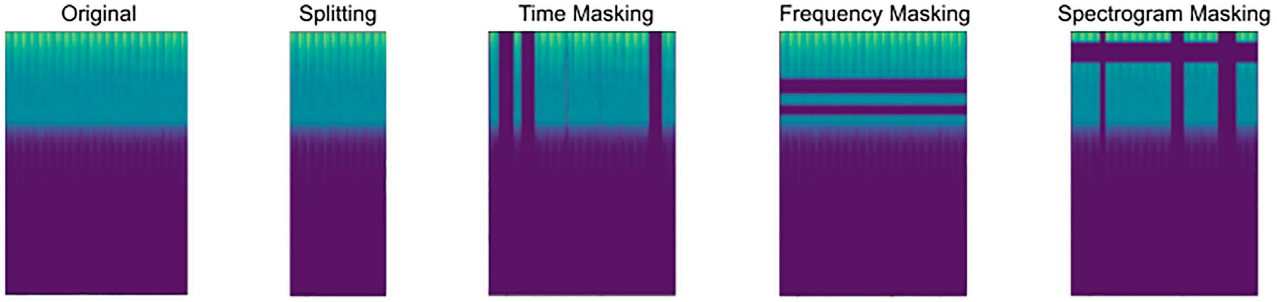

Figure 1. Examples of spectrogram augmentation strategies
Note that in masking routines, masking is only applied to the only central audio sample, not left or right padding.

comparable or improved performance compared to the baseline at both 10% and 100% levels, with baseline performance of 0.512 (95% CI: 0.484, 0.536) and 0.516 (95% CI: 0.463, 0.559), respectively. Of the schemes, spectrogram masking provides the most pronounced increase in performance relative to the baseline with AUCs of 0.652 (95% CI: 0.597, 0.704) and 0.659 (95% CI: 0.600, 0.716), with frequency masking and time masking performing comparably. At both 10% and 100% data levels, masking along the time dimension only provides significant improvement compared to spectrogram masking and splitting ($p < 0.001$ and $p < 0.001$, respectively), with AUCs of 0.643 (95% CI: 0.598, 0.695) and 0.654 (95% CI: 0.595, 0.715) compared with AUCs of 0.533 (95% CI: 0.498, 0.568) and 0.609 (95% CI: 0.549, 0.668). Statistical significance is measured using a two-tailed two-sample paired t test.

Evaluating initializations via end-to-end fine-tuning

In terms of weight initializations, of the augmentation strategies, the best performance is provided by spectrogram masking with AUCs of 0.633 (95% CI: 0.582, 0.697) and 0.691 (95% CI: 0.628, 0.758) at 10% and 100%, respectively. This is comparable to performance with supervised training, which achieves 10% and 100% AUCs of 0.628 (95% CI: 0.585, 0.730) and 0.690 (95% CI: 0.636, 0.754).

Figure 3 shows experimental test results for lung sound classification.

Incorporating sample metadata for lung sounds

We further explore the effect of using metadata-based contrastive views. Notably, we do not use augmentations in this setup to isolate the effects of metadata selection compared to established field methodologies (augmentations). In our incorporation of metadata, we consider explicit selection criteria for negative pairs as well as positive pairs. This is not part of the simple framework for contrastive learning of visual representations (SimCLR) framework. The available metadata consist of whether the patient is a child or adult (which we refer to as "age"), the patient's sex, and the recording location (trachea, anterior left, anterior right, posterior left, posterior right, lateral left, or lateral right).

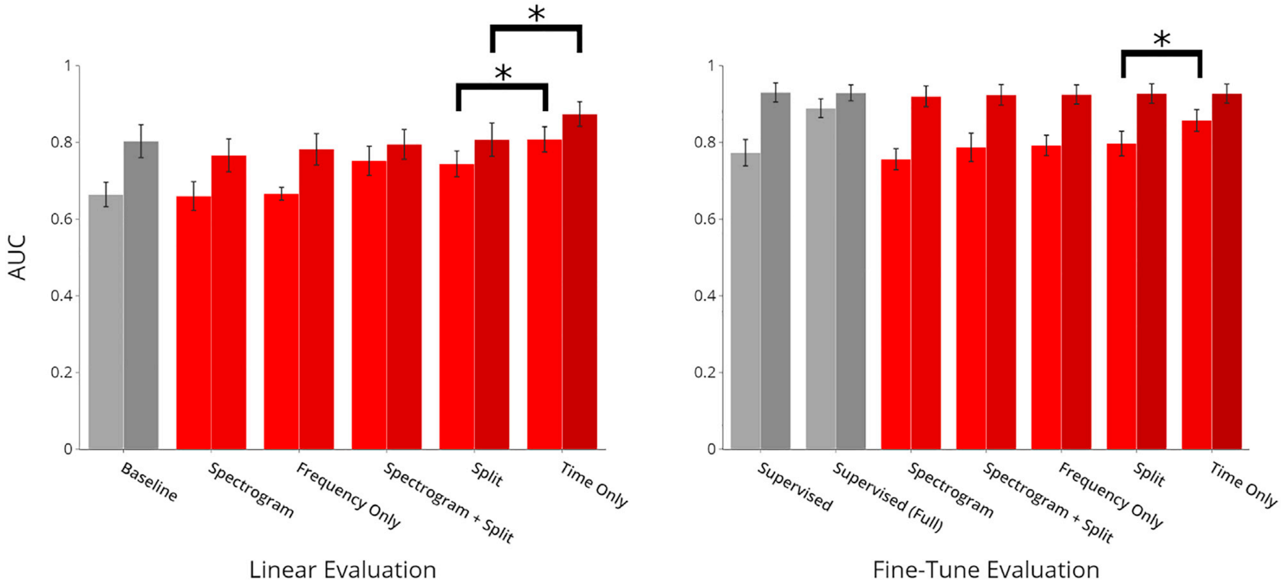

Figure 2. Experimental results for heart sound classification
AUCs are reported with 95% confidence intervals (CIs) (error bars). Asterisks indicate statistically significant differences ($p < 0.001$). Performance at 10% and 100% training data levels are presented for each learning scheme. All contrastive schemes match or surpass baselines for linear evaluation at 10% and 100%. All contrastive schemes match or surpass baseline performance at 10% with performance saturation for 100% in fine-tune evaluation. See also Table S1.

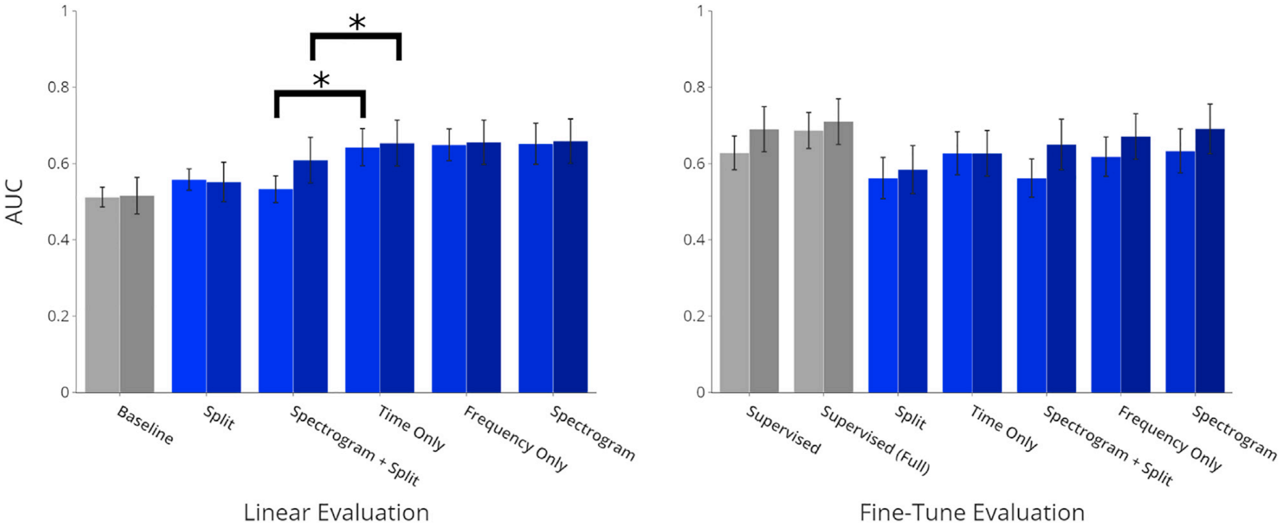

Figure 3. Experimental results for lung sound classification

AUCs are reported with 95% confidence intervals (error bars). Performance at 10% and 100% training data levels are presented for each learning scheme. Asterisks indicate statistically significant differences ($p < 0.001$). All contrastive schemes match or surpass baselines for linear evaluation at 10% and 100%. For fine-tune evaluation, spectrogram masking provides the best performance, comparable with supervised training at both 10% and 100%. See also Table S2.

We speculate that these factors are either correlated with presence of disease (age and sex[17]) or can provide insight into spatial information for improved learning (recording location[18]).

We construct contrastive schemes by selecting from these metadata features to create rules for positive and negative pairs. Starting with age, we consider positive pair selection as well as negative pair selection, abbreviated as "pos. sim. age" and "neg. sim. age," respectively. For locations, we consider positive selection for the same location (pos. same loc.) as well as positive selection for different locations (pos. dif. loc.) to determine the importance of spatial association. We also consider the combination of positive and negative selection on location with pos. same loc./neg. same loc. We finally consider choosing negative pairs by sex (neg. sim. sex) and choosing negative pairs by age and sex (neg. sim. age + sex).

In terms of representations, we see marked differences between different selection policies. Forming negative pairs with respect to age bands (adult versus child) provides significant improvement (AUCs of 0.788 [95% CI: 0.739, 0.834] and 0.773 [95% CI: 0.718, 0.82] for 10% and 100%, respectively) over other contrastive schemes, other than neg. sim. age + sex. Statistical significance is measured using a two-tailed two-sample paired t test compared with neg. sim. sex with $p < 0.001$ and $p < 0.001$ at both 10% and 100%. The further selection of sex along with age provides an additional increase in performance (AUCs of 0.854 [95% CI: 0.823, 0.882] and 0.863 [95% CI: 0.834, 0.890] for 10% and 100%, respectively) over neg. sim. age. Statistical significance is measured using a two-tailed two-sample paired t test with $p < 0.001$ and $p < 0.001$ at both 10% and 100%.

Observing performance for weight initializations, we see corresponding trends to that of representations. Negative selection for age bands provides improvement over other demographic methods (AUCs of 0.782 [95% CI: 0.737, 0.830] and 0.785 [95% CI: 0.734, 0.838] for 10% and 100%, respectively). We further note significant performance significant improvement at 10% and 100% ($p < 0.001$ and $p < 0.001$, respectively) when compared with the supervised baseline. Statistical significance is measured using a two-tailed two-sample paired t test. The addition of negative selection by sex provides further improvement (AUCs of 0.822 [95% CI: 0.782, 0.854] and 0.842 [95% CI: 0.803, 0.876] for 10% and 100%, respectively) over neg. sim. age. Statistical significance is measured using a two-tailed two-sample paired t test with $p < 0.001$ and $p < 0.001$ at both 10% and 100%.

Further analyzing trends between selection groups, we find that positive selection in general has less efficacy than negative selection. For representations, for example, we observe AUCs of 0.663 (95% CI: 0.610, 0.713) and 0.674 (95% CI: 0.618, 0.726) for 10% and 100%, respectively for pos. sim. age. Using a two-sample paired t test against neg. sim. age, we observe a statistically significant difference, with $p < 0.001$ for both 10% and 100%. This result may be due to the fact that positive selection by age matches up multiple samples that do not share true labels. The SSL with linear evaluation outperforms SSL with fine-tuning. We hypothesize that this resulted from overfitting of the fine-tuning method on the data, which may be expected given the small number of samples in the train set. We expect that fine-tuning would perform better given more training data. Furthermore, both representation and initialization experimental performances show that age is a more valuable metadata tool. Given that there are strong correlations between age and presence of respiratory disease,[19,20] it follows that selection by age provides more difficult pairs to differentiate compared with other metadata features. Finally, we remark that the combination of these factors enables better performance compared with either of the factors alone (age and sex, 0.854 [95% CI: 0.823, 0.805]; age alone, 0.788 [95% CI: 0.739, 0.834]; sex only, 0.723 [95% CI: 0.675, 0.770]).

Figure 4 presents results for contrastive self-supervised methodologies based on sample metadata.

CellPress
OPEN ACCESS

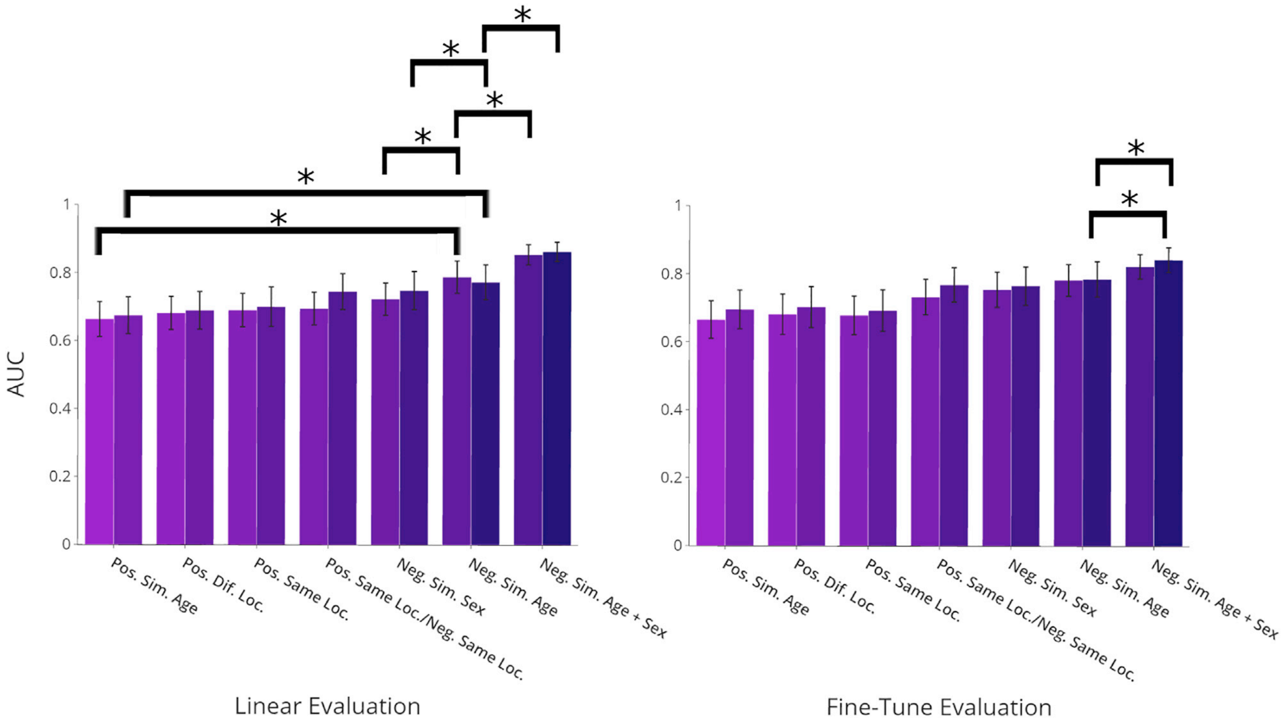

Figure 4. Experimental results for lung sound classification with incorporated sample metadata
AUCs are reported with 95% confidence intervals (error bars). Asterisks indicate statistically significant differences ($p < 0.001$). Performance at 10% and 100% training data levels are presented for each learning scheme. In both linear and fine-tune evaluation setups, there are marked differences between contrastive schemes, with the negative pair selection of age and sex providing superior performance in both setups. See also Table S3.

DISCUSSION

We introduce a contrastive learning framework that utilizes unlabeled data with associated metadata to select positive and negative pairs. We first compare different augmentation methods for sounds. We find that time masking and spectrogram masking are the best performing methods for heart sounds and for lung sounds, respectively. These results demonstrate that augmentation methods perform differently in different contexts, and it is important to optimize contrastive learning frameworks according to the type of data. We then describe the contrastive learning framework utilizing metadata to select positive and negative pairs, rather than augmentation methods. Results in lung sounds show that negative pair selection based on age improve downstream lung sound diagnosis tasks the most, followed by those based on sex. This is in accordance with clinical experience that lung diseases correlate with age and sex.[21,22] These results show that metadata, especially when used in correlation with domain expertise, can be a powerful way to select positive and negative pairs in contrastive learning and that leveraging such information can further improve representations learned from contrastive learning.

Limitation in labeled data is a bottleneck for many applications of supervised learning. Self-supervised learning can leverage unlabeled data to learn general meaningful representations and use labeled data for fine-tuning. This approach has been applied with success across domains, including for natural language processing[23–25] and computer vision[1,2,26,27] tasks. In the domain of audio signals, most studies have focused on speech recognition tasks, showing better results with less labeled data in downstream tasks, such as predicting characters,[28] spoken language modeling without text,[29] and speaker recognition.[30]

Contrastive learning has been used to leverage unlabeled medical data to improve performance on downstream tasks.[18,31] For example, a study used contrastive learning to explore patient-level shared context in electrocardiogram signals across different temporal and spatial segments.[18] Another example of contrastive learning for time-series data builds upon the SimCLR framework to learn channel-specific features in electroencephalograms.[31] These two studies focus on representations for either individual patients or a channel of signal, while our study focuses on using shared characteristics between different patients to find representations for a group of patients, potentially leading to greater generalizability. Another study uses contrastive learning for medical imaging, leveraging patient metadata, including same patient, same imaging study, or same laterality to select positive pairs.[8] Contrary to our results, this study saw no benefit in using patient metadata to select negative pairs, which may be due to high similarities among chest images compared to sound recordings.

In conclusion, our work presents a contrastive learning framework that is able to leverage associated information for pair selection despite imbalanced source datasets. We demonstrate its application in audio processing in the medical context using heart and lung sounds and related clinical data for selecting contrastive pairs across different segments and sources. The performance of our model decreases with external validation

using heart and lung sounds collected at a different clinical sites. The suboptimal generalizability could be potentially due to the limited size of the training data. During training, the model may not have learned features that are generalizable across different populations. One potential improvement that can be made could be pre-training with a larger dataset with a wide variety of sounds. Batch effects may explain the suboptimal generalizability given that the lung and heart sounds from training datasets and external validation datasets were collected using different stethoscopes at different sites. Carefully tuning mixtures of metadata and augmentation-based contrastive methodologies may prove to be fruitful future explorations as could systematically exploring the effect of imbalanced pre-training datasets. Our study provides useful insight across domains beyond healthcare, as associated information can also be used in other contexts. Audio augmentation methods continue to develop and become more and more versatile.[32] As shown by our results and previous work,[33,34] the best performing augmentation method may depend on the type of signal data used. Leveraging metadata rather than solely relying on augmentation can be a powerful tool for generating positive and negative in contrastive learning in various types of audio and signal data. With advancement of databases that include multiple types of information on each patient, including omics and medical images, the power of contrastive learning could potentially be augmented, and the connection between metadata, lung and heart sounds, and diseases could potentially be better elucidated. In addition, the contrastive learning approach with metadata we outlined could be tried with multiple types of metadata to broaden the application of our current methods. However, further research in this area is limited by a general lack of interlinked multi-type datasets, an area of focus for healthcare artificial intelligence research.

EXPERIMENTAL PROCEDURES

Resource availability

Lead contact

Further information and requests for resources and reagents should be directed to and will be fulfilled by the lead contact, Pranav Rajpurkar (pranav_rajpurkar@hms.harvard.edu).

Materials availability

This study did not generate new unique reagents.

Data and code availability

This paper analyzes existing, publicly available data. The respiratory sound database can be found at https://bhichallenge.med.auth.gr/. The PhysioNet Heart Challenge can be found at https://physionet.org/content/challenge-2016/1.0.0/#files. The DOI for the external validation dataset is https://doi.org/10.17632/jwyy9np4gv.3.

All original code has been deposited at Zenodo under https://doi.org/10.5281/zenodo.5715686 and is publicly available as of the date of publication.

Any additional information required to reanalyze the data reported in this paper is available from the lead contact upon request.

Data

We consider two datasets for training and internal testing; for lung sounds, we use the respiratory sound database,[35] and for heart sounds, we use the PhysioNet Heart Challenge.[16] We use another dataset for external validation: lung sounds recorded at a different clinical site.[36]

For the PhysioNet Heart Challenge, we consider the task of classifying normal sounds versus abnormal sounds. The dataset consists of 3,240 samples (2,575 normal and 665 abnormal), which were center-padded and cropped to a consistent size. 400 examples each were reserved for testing and validation with a one-to-one ratio of classes in the samples. The remaining 2,440 examples represent the pre-train set, of which 400 examples were taken for fine-tuning (again with a one-to-one ratio of classes). The pre-train set is used for contrastive learning with the fine-tune set used for downstream training for representation/initialization tasks. The test set is finally used to evaluate trained models.

For the respiratory sound database, we modify the original task from multi-class diagnosis to the same binary classification problem from above (normal versus abnormal) due to limitations in data independence between samples, as there is only one asthma patient. The original classes presented are healthy, chronic obstructive pulmonary disease (COPD), upper respiratory tract infection (URTI), bronchiectasis, bronchiolitis, pneumonia, lower respiratory tract infection, and asthma, with some classes having only one patient (asthma). As such, we shift the task from differentiating between multiple signal classes to differentiating between abnormal and normal signals. However, we do test if the pre-trained models are able to extract features differentiating the original classes; these extracted features were plotted using a 50-dimensional principal component analysis dimensionality reduction followed by a two-factor t-distributed stochastic neighbor embedding as shown in Figure 5, with spectrogram and neg. sim. age + sex on the left and right columns, respectively. We present perplexity levels of 5, 25, and 50. The differentiation between COPD- and URTI-labeled samples, as well as the general clustering of pneumonia samples, indicates that the models do in fact learn to implicitly differentiate these different signal classes within the larger abnormal class.

The respiratory sound database contains incomplete demographic information and metadata including age, sex, height, weight, and body mass index, as well as recording location. Missing patient demographics information was imputed using multiple imputation by chained equations. Notably, one patient does not have any associated demographic data, so data were imputed by averaging across all other patients.

The lung sound external validation dataset includes lung sounds and related demographics information about patients. As above, we modify the original task to the binary classification of normal versus abnormal. The original classes are normal, asthma, pneumonia, COPD, bronchitis, heart failure, lung fibrosis, and pleural effusion. The dataset contains 112 recordings, each from a unique patient.

Tasks

We test pre-training schemes using the downstream tasks of representations and initializations. For comparing representations, we consider the task of passing inputs through an encoder model (pre-trained with self-supervision, then freezing the base network parameters) to generate a lower-dimensional latent space and then training a linear evaluator with the transformed data. We compare results against a linear model directly taking in the flattened spectrogram. This serves as a benchmark to evaluate the efficacy of the encoder backbone and pre-training methodology at extracting relevant features.

For comparing initializations, we consider the task of using pre-trained weights to initialize a classification model constructed by appending a two-layer SSL evaluator and then fine-tuning all of the model parameters with labeled data. We compare against an equivalent model but with randomly initialized weights rather than the pre-trained ones, which serves to show the effectiveness of pre-training data as a way to jumpstart supervised learning and the overall final performance of the model.

Contrastive learning

With contrastive learning, we build robust vector representations using unlabeled or weakly labeled data. When considering the overall embedding space defined by these learned vectors, we wish for these representations to be close for similar inputs and apart for dissimilar inputs. We then use the learned vectors/pre-trained model for downstream tasks, such as the diagnosis classification as in this study.

To obtain these vectors, we apply and extend the self-supervised methodology employed in SimCLRv2, as shown in Figure 6. As our inputs, we consider views $\tilde{x}_i$ and $\tilde{x}_j$, generated by applying randomized augmentations or selecting against recordings from the same sample (sample metadata experiments). The pair is then encoded (using a Resnet-18 backbone) to vector representations h_i and h_j, respectively. The representations are then passed through a linear

CellPress
OPEN ACCESS

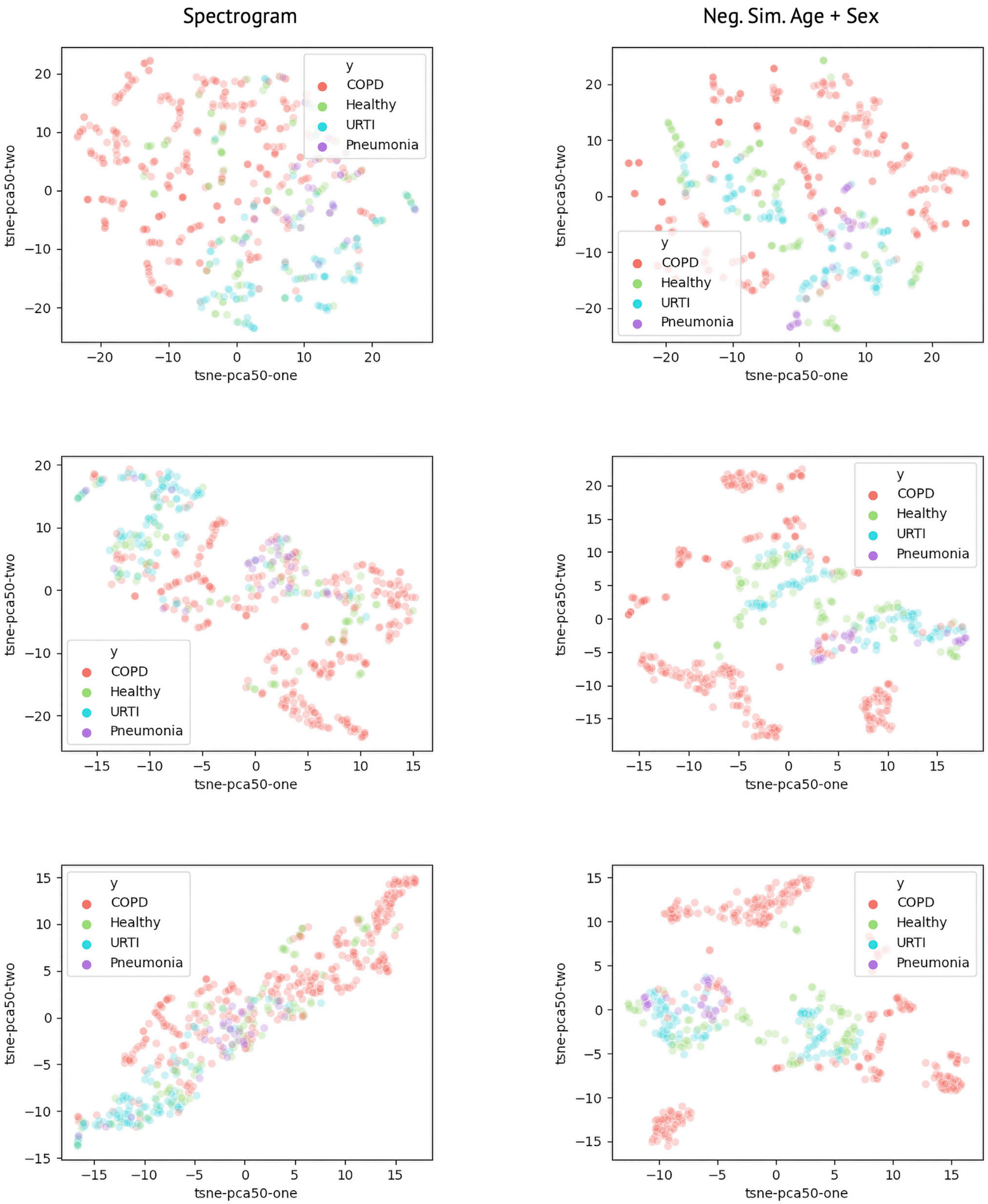

Figure 5. Two component t-SNE plot for dimensionality reduced embedding vectors for lung sounds

Spectrogram and neg. sim. age + sex are on the left and right columns at perplexity levels of 5, 25, and 50 from the top down. The relative clustering of each of the abnormal classes (COPD, URTI, and Pneumonia) indicates that the models learn to differentiate these signal classes even without these explicit labels.

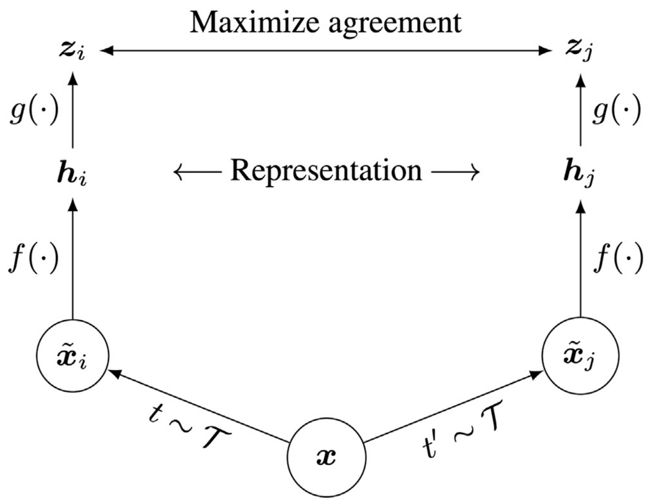

Figure 6. SimCLR learning framework
For a given input x, we apply random transforms to produce x_i and x_j. We then use encoding function f to produce representations that are projected by projection head g to get z_i and z_j. During contrastive learning, we maximize the agreement between z_i and z_j.

projection head to reduce dimensionality. This gives us z_i and z_j, and we wish to maximize the similarity of this pair. By maximizing this agreement, we encapsulate the complexity of the unlabeled data for use as representations, as well as using pre-training the encoder as an initialization point for further fine-tuning.

We measure agreement for the singular positive pair (z_i and z_j), using normalized temperature-scaled cross entropy (NT-Xent) loss, as shown in Equation 1. NT-Xent loss measures the ratio of the similarity between the positive pair to the sum of similarities across all possible pairs, positive and negative, in the batch represented by $A(i)$.

$$\ell = -\log\left\{\frac{\exp(\mathrm{sim}(\mathbf{z_i}, \mathbf{z_j})/T)}{\sum_{a \in A(i)} \exp\left(\mathrm{sim}(\mathbf{z_i}, \mathbf{z_a})/T\right)}\right\}$$

Models

We utilize a ResNet-18 backbone to generate encodings in the latent space of length N. For pre-training, these latent encodings are cast through a projection head to a dimensionality of 256. For downstream evaluation, this projection head is removed and replaced with a single linear layer (with encoder weights frozen) and an SSL online evaluator for representation and initialization fine-tuning, respectively, as shown in Figure 7. In linear evaluation, the linear layer takes in a length N feature vector and outputs a single output passed through sigmoid activation. In the SSL evaluator, a length N feature vector is passed through a linear layer without output length N, and batch normalization is then applied followed by ReLU activation. The subsequent linear layer provides a single output passed through sigmoid activation.

Learning framework modifications for incorporation of metadata

The selection criteria for metadata, as part of an SSL method, only makes use of available metadata (age, gender, and location), and does not use the annotations (diagnoses) associated with any of the data points. We generate contrastive views by selecting two independent samples for a given patient subject to the specified condition on positive pairs. We note that the default SimCLR setup does not support negative pair selection by default, which becomes relevant for our metadata studies. For the following setups, we modify batch generation so that all samples within the batch share the negative selection trait: pos. same loc./neg. same loc., neg. sim. age, neg. sim. sex, and neg. sim. age + sex. For pos. sim. age, we adapt the supervised contrastive learning methodology, using the age bands as weak labels. The dataset for heart and lung sounds did not provide other types of metadata. In our example, age is a very generalizable metadata item for positive and negative pair selection, given that age plays a key role in many medical diseases.

We hypothesize that the selection of negative pairs by patient and audio metadata provides a stronger pre-training task. If we consider Equation 1, with negative pairs that are more similar to the given example, the sum in the denominator increases, leading to an increase in the sample error. Therefore, the model must work harder to differentiate these pairs from positive ones, improving downstream performance.

Supervised contrastive learning

Supervised contrastive learning leverages labels to provide better contrastive strategies.[37] In this study, instead of true labels (classifications), we utilize soft labels sourced from metadata. In this setup, models of the same class are considered positive pairs, while those of different classes are negative. This method therefore introduces additional positive pairs into the fold. We modify the loss function from NT-Xent to incorporate these additional pairs, as shown in Equation 2. In this modified setup, for each of the positive pairs, $P(i)$, we take the ratio between its similarity and the sum of the similarities of all pairs, positive and negative, as represented by $A(i)$. We then average these scores before taking the negative logarithm.

$$L = -\log\left\{\frac{1}{|P(i)|}\sum_{p \in P(i)} \frac{\exp(\mathrm{sim}(\mathbf{z_i}, \mathbf{z_j})/T)}{\sum_{a \in A(i)} \exp\left(\mathrm{sim}(\mathbf{z_i}, \mathbf{z_a})/T\right)}\right\}$$

Model training and hyperparameters

We train all models with a batch size of 16 and a learning rate of 1E-5. For pre-training, we use an L2 penalty factor of 1E-6, and for downstream training we use an L2 penalty of 1E-2. Unless otherwise noted, linearly evaluated models (representations) are trained for 5,000 epochs, and fine-tuned models (initializations) are trained for 25 epochs. For linear baselines, we train for 500 epochs for heart data and 1,000 epochs for lung data, at which point convergence is achieved. For the 10% data level, 20 models were trained in parallel; for the 100% data level, 5 models were trained in parallel. Testing performance is evaluated using AUROC, using bootstrapped CIs with 1,000 replicates for each model.

Data processing

Both the lung and heart datasets contain samples of vastly varying lengths. In order to standardize, samples are cropped/padded at a threshold of the 75th quartile of recording lengths. Cropping/padding is done so that recordings are aligned to the center (the middle of the recording is the middle of the spectrogram). Furthermore, when masking data, we apply transformations to only data regions with five bands in the time dimension and two bands in the frequency dimension. We chose a target of 50% data masking on average and manually checked samples to ensure features of interest were not lost.

SUPPLEMENTAL INFORMATION

Supplemental information can be found online at https://doi.org/10.1016/j.patter.2021.100400.

ACKNOWLEDGMENTS

We thank Hari Sowrirajan and Ryan Chi for proof-reading this article.

AUTHOR CONTRIBUTIONS

Conceptualization ideas, P.N.S., S.S., and P.R.; methodology, P.N.S., P.R.S., S.S., and P.R.; software programming, P.N.S. and P.R.S.; validation verification, P.N.S., S.S., and P.R.; formal analysis, P.N.S. and S.S.; resources, A.N. and P.R.; data curation, P.N.S. and S.S.; writing – original draft, P.N.S., S.S., and P.R.S.; writing – review & editing, P.N.S., S.S., P.R., and A.N.; visualization preparation, P.N.S., S.S., and P.R.S.; supervision, A.N. and P.R.; project administration, A.N. and P.R.; funding acquisition, A.N.

CellPress
OPEN ACCESS

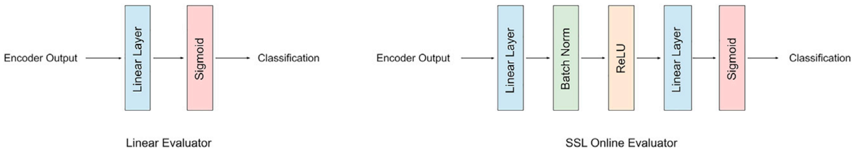

Figure 7. Block diagrams illustrating pipelines for evaluators used for representation (linear) and initialization (SSL online evaluator), respectively

Note that in the linear pipeline, the encoder parameters are frozen; they are not updated during fine-tuning. On the other hand, the SSL online evaluator is part of an end-to-end pipeline where the encoder parameters are updated alongside the evaluator.

DECLARATION OF INTERESTS

The authors declare no competing interests.

Received: June 16, 2021
Revised: July 29, 2021
Accepted: November 5, 2021
Published: December 7, 2021

REFERENCES

1. Le-Khac, P.H., Healy, G., and Smeaton, A.F. (2020). Contrastive Representation Learning: A Framework and Review (IEEE Access).
2. Chen, T., Kornblith, S., Norouzi, M., and Hinton, G. (2020). A simple framework for contrastive learning of visual representations. In PMLR), pp. 1597–1607.
3. Jiao, J., Droste, R., Drukker, L., Papageorghiou, A.T., and Noble, J.A. (2020). Self-supervised representation learning for ultrasound video. Proc. IEEE Int. Symp. Biomed. Imaging *2020*, 1847–1850.
4. Al-Tahan, H., and Mohsenzadeh, Y. (2021). CLAR: contrastive learning of auditory representations. In International Conference on Artificial Intelligence and Statistics (PMLR), pp. 2530–2538.
5. Ferraro, A., Favory, X., Drossos, K., Kim, Y., and Bogdanov, D. (2021). Enriched music representations with multiple cross-modal contrastive learning. IEEE Signal Process. Lett. *28*, 733–737.
6. Tian, Y., Sun, C., Poole, B., Krishnan, D., Schmid, C., and Isola, P. (2020). What makes for good views for contrastive learning? Adv. Neural Inf. Process. Syst. *33*, 6827–6839.
7. Hu, S.-Y., Wang, S., Weng, W.-H., Wang, J., Wang, X., Ozturk, A., Li, Q., Kumar, V., and Samir, A.E. (2020). Self-supervised pretraining with DICOM metadata in ultrasound imaging. In Machine Learning for Healthcare Conference (PMLR), pp. 732–749.
8. Vu, Y.N.T., Wang, R., Balachandar, N., Liu, C., Ng, A.Y., and Rajpurkar, P. (2021). MedAug: contrastive learning leveraging patient metadata improves representations for chest X-ray interpretation. arXiv:2102.10663 [cs, eess].
9. Elouni, J., Ellouzi, H., Ltifi, H., and Ayed, M.B. (2020). Intelligent health monitoring system modeling based on machine learning and agent technology. Multiagent Grid Syst. *16*, 207–226.
10. Wanyan, T., Honarvar, H., Jaladanki, S.K., Zang, C., Naik, N., Somani, S., De Freitas, J.K., Paranjpe, I., Vaid, A., Miotto, R., et al. (2021). Contrastive learning improves critical event prediction in COVID-19 patients. arXiv:2101.04013 [cs].
11. Kumar, R., Weill, E., Aghdasi, F., and Sriram, P. (2019). Vehicle re-identification: an efficient baseline using triplet embedding. arXiv:1901.01015 [cs].
12. Halbersberg, D., Hillel, A.B., Mendelson, S., Koster, D., Karol, L., and Lerner, B. (2020). Transfer learning of photometric phenotypes in agriculture using metadata. arXiv:2004.00303 [cs].
13. Borgholt, L., Tax, T.M.S., Havtorn, J.D., Maaløe, L., and Igel, C. (2021). On scaling contrastive representations for low-resource speech recognition. arXiv:2102.00850 [cs, eess].
14. Aberdam, A., Litman, R., Tsiper, S., Anschel, O., Slossberg, R., Mazor, S., Manmatha, R., and Perona, P. (2020). Sequence-to-sequence contrastive learning for text recognition. arXiv:2012.10873 [cs].
15. Rocha, B., Filos, D., Mendes, L., Vogiatzis, I., Perantoni, E., Kaimakamis, E., Natsiavas, P., Oliveira, A., Jácome, C., and Marques, A. (2017). A Respiratory Sound Database for the Development of Automated Classification (Springer), pp. 33–37.
16. Liu, C., Springer, D., Li, Q., Moody, B., Juan, R.A., Chorro, F.J., Castells, F., Roig, J.M., Silva, I., Johnson, A.E.W., et al. (2016). An open access database for the evaluation of heart sound algorithms. Physiol. Meas. *37*, 2181–2213.
17. Groeneveld, J.M., Ballering, A.V., van Boven, K., Akkermans, R.P., Olde Hartman, T.C., and Uijen, A.A. (2020). Sex differences in incidence of respiratory symptoms and management by general practitioners. Fam. Pract. *37*, 631–636.
18. Kiyasseh, D., Zhu, T., and Clifton, D.A. (2020). CLOCS: contrastive learning of cardiac signals across space, time, and patients. arXiv:2005.13249 [cs, eess, stat].
19. Lowery, E.M., Brubaker, A.L., Kuhlmann, E., and Kovacs, E.J. (2013). The aging lung. Clin. Interv. Aging *8*, 1489–1496.
20. Zar, H.J., and Ferkol, T.W. (2014). The global burden of respiratory disease-impact on child health. Pediatr. Pulmonol *49*, 430–434.
21. Meiners, S., Eickelberg, O., and Königshoff, M. (2015). Hallmarks of the ageing lung. Eur. Respir. J. *45*, 807–827.
22. Gross, V., Dittmar, A., Penzel, T., Schuttler, F., and Von Wichert, P. (2000). The relationship between normal lung sounds, age, and gender. Am. J. Respir. Crit. Care Med. *162*, 905–909.
23. Rethmeier, N., and Augenstein, I. (2021). A primer on contrastive pretraining in language processing: methods, lessons learned and perspectives. arXiv:2102.12982.
24. Giorgi, J.M., Nitski, O., Bader, G.D., and Wang, B. (2020). DeCLUTR: deep contrastive learning for unsupervised textual representations. arXiv:2006.03659.
25. Chen, X., Xie, X., Bi, Z., Ye, H., Deng, S., Zhang, N., and Chen, H. (2021). Disentangled contrastive learning for learning robust textual representations. arXiv:2104.04907.
26. Deng, Y., Yang, J., Chen, D., Wen, F., and Tong, X. (2020). Disentangled and controllable face image generation via 3D imitative-contrastive learning. In, pp. 5154–5163.
27. Chaitanya, K., Erdil, E., Karani, N., and Konukoglu, E. (2020). Contrastive learning of global and local features for medical image segmentation with limited annotations. arXiv:2006.10511 [cs, eess, stat].
28. Baevski, A., Zhou, Y., Mohamed, A., and Auli, M. (2020). NeurIPS 2020: wav2vec 2.0: a framework for self-supervised learning of speech representations. In Advances in Neural Information Processing Systems, *33*, H. Larochelle, M. Ranzato, R. Hadsell, M.F. Balcan, and H. Lin, eds. (Curran Associates), pp. 12449–12460.
29. Nguyen, T.A., de Seyssel, M., Rozé, P., Rivière, M., Kharitonov, E., Baevski, A., Dunbar, E., and Dupoux, E. (2020). The zero resource speech benchmark 2021: metrics and baselines for unsupervised spoken language modeling. arXiv:2011.11588 [cs, eess].
30. Huh, J., Heo, H.S., Kang, J., Watanabe, S., and Chung, J.S. (2020). Augmentation adversarial training for self-supervised speaker recognition. arXiv:2007.12085 [cs, eess].
31. Mohsenvand, M.N., Izadi, M.R., and Maes, P. (2020). Contrastive representation learning for electroencephalogram classification. In

Proceedings of the Machine Learning for Health NeurIPS Workshop (PMLR), pp. 238–253.

32. Wei, S., Zou, S., Liao, F., and Lang, W. (2020). A comparison on data augmentation methods based on deep learning for audio classification. J. Phys. Conf. Ser. *1453*, 012085.
33. Nasiri, A., and Hu, J. (2021). SoundCLR: contrastive learning of representations for improved environmental sound classification. arXiv:2103.01929 [cs, eess].
34. Kharitonov, E., Rivière, M., Synnaeve, G., Wolf, L., Mazaré, P.-E., Douze, M., and Dupoux, E. (2020). Data augmenting contrastive learning of speech representations in the time domain. arXiv:2007.00991 [cs, eess].
35. Marsh Respiratory Sound Database. https://kaggle.com/vbookshelf/respiratory-sound-database.
36. Fraiwan, M., Fraiwan, L., Khassawneh, B., and Ibnian, A. (2021). A dataset of lung sounds recorded from the chest wall using an electronic stethoscope. Data in Brief *35*, 106913.
37. Khosla, P., Teterwak, P., Wang, C., Sarna, A., Tian, Y., Isola, P., Maschinot, A., Liu, C., and Krishnan, D. (2020). Supervised contrastive learning.arXiv:2004.11362

Patterns, Volume *3*

Supplemental information

Contrastive learning of heart and lung sounds for label-efficient diagnosis

Pratham N. Soni, Siyu Shi, Pranav R. Sriram, Andrew Y. Ng, and Pranav Rajpurkar

Supplementary Tables

Table S1: Supplementary data for Figure 2.

		AUC (95% CI)	
Type	Augmentation	10% fraction	100% fraction
No Encoder Baseline	No Augmentation	0.664 (0.630, 0.694)	0.803 (0.755, 0.841)
Supervised Learning	No Augmentation	0.773 (0.737, 0.806)	**0.930 (0.904, 0.954)**
Supervised Learning (full)	No Augmentation	**0.889 (0.865, 0.913)**	0.929 (0.907, 0.949)
	Spectrogram	0.756 (0.728, 0.783)	0.920 (0.894, 0.948)
	Split	0.797 (0.768, 0.832)	**0.927 (0.901, 0.952)**
	Spectrogram + Split	0.787 (0.753, 0.827)	0.924 (0.898, 0.952)
	Frequency Only	0.792 (0.764, 0.817)	0.925 (0.900, 0.950)
Self-supervised Learning (finetune)	Time Only	**0.857 (0.828, 0.885)**	**0.927 (0.901, 0.951)**
	Spectrogram	0.660 (0.619, 0.694)	0.766 (0.719, 0.805)
	Split	0.744 (0.711, 0.778)	0.807 (0.763, 0.850)
	Spectrogram + Split	0.752 (0.715, 0.791)	0.795 (0.759, 0.836)
	Frequency Only	0.666 (0.663, 0.696)	0.782 (0.736, 0.818)
Self-supervised Learning (linear)	Time Only	**0.808 (0.772, 0.838)**	**0.874 (0.841, 0.905)**

Table S2: Supplementary data for Figure 3.

		AUC (95% CI)	
Type	Augmentation	10% fraction	100% fraction
No Encoder Baseline	No Augmentation	0.512 (0.484, 0.536)	0.516 (0.463, 0.559)

Supervised Learning	No Augmentation	0.628 (0.585, 0.673)	0.690 (0.636, 0.754)
Supervised Learning (full)	No Augmentation	**0.687 (0.636, 0.730)**	**0.710 (0.655, 0.774)**
	Spectrogram	**0.633 (0.582, 0.697)**	**0.691 (0.628, 0.758)**
	Split	0.562 (0.510, 0.618)	0.584 (0.519, 0.645)
	Spectrogram + Split	0.562 (0.514, 0.615)	0.650 (0.586, 0.718)
	Frequency Only	0.618 (0.568, 0.671)	0.671 (0.615, 0.734)
Self-supervised Learning (finetune)	Time Only	0.627 (0.572, 0.685)	0.627 (0.571, 0.690)
	Spectrogram	**0.652 (0.597, 0.704)**	**0.659 (0.600, 0.716)**
	Split	0.558 (0.528, 0.584)	0.552 (0.499, 0.603)
	Spectrogram + Split	0.533 (0.498, 0.568)	0.609 (0.549, 0.668)
	Frequency Only	0.649 (0.601, 0.684)	0.656 (0.597, 0.713)
Self-supervised Learning (linear)	Time Only	0.643 (0.598, 0.695)	0.654 (0.595, 0.715)

Table S3: Supplementary data for Figure 4.

		AUC (95% CI)	
Type	Pair Selection Method	10% fraction	100% fraction
	Pos. Same Loc.	0.678 (0.621, 0.735)	0.692 (0.635, 0.758)
	Pos. Dif. Loc.	0.681 (0.626, 0.745)	0.702 (0.643, 0.764)
	Pos. Same Loc./Neg. Same Loc.	0.732 (0.679, 0.783)	0.768 (0.717, 0.818)
Self-supervised Learning (finetune)	Pos. Sim. Age	0.665 (0.610, 0.720)	0.695 (0.638, 0.752)

	Neg. Sim. Age	0.782 (0.737, 0.830)	0.785 (0.734, 0.838)
	Neg. Sim. Sex	0.754 (0.703, 0.806)	0.765 (0.711, 0.824)
	Neg. Sim. Age + Sex	**0.822 (0.782, 0.854)**	**0.842 (0.803, 0.876)**
	Pos. Same Loc.	0.690 (0.638, 0.736)	0.700 (0.641, 0.757)
	Pos. Dif. Loc.	0.681 (0.635, 0.733)	0.689 (0.636, 0.747)
	Pos. Same Loc./Neg. Same Loc.	0.695 (0.646, 0.742)	0.745 (0.691, 0.796)
	Pos. Sim. Age	0.663 (0.610, 0.713)	0.674 (0.618, 0.726)
	Neg. Sim. Age	0.788 (0.739, 0.834)	0.773 (0.718, 0.820)
	Neg. Sim. Sex	0.723 (0.675, 0.770)	0.748 (0.692, 0.804)
Self-supervised Learning (linear)	Neg. Sim. Age + Sex	**0.854 (0.823, 0.882)**	**0.863 (0.834, 0.890)**
